# Coronary Slow Flow Phenomenon: A Narrative Literature Review

**DOI:** 10.7759/cureus.88657

**Published:** 2025-07-24

**Authors:** Khudheeja A Ahmed, Wasifuddin Syed, Juwayria A Ahmed, Mohammed Habeeb Ahmed

**Affiliations:** 1 College of Osteopathic Medicine, Touro University California, Vallejo, USA; 2 Department of Medicine, Edward Via College of Osteopathic Medicine, Monroe, USA; 3 Department of Research, KAAJ Healthcare, San Jose, USA; 4 Department of Cardiology, KAAJ Healthcare, San Jose, USA

**Keywords:** coronary slow flow, csf prognosis, csf treatment, endothelial dysfunction, syndrome y

## Abstract

Coronary slow flow (CSF) is understood to be a clinical condition during which angiographic findings show the slowed filling of contrast in the coronary vasculature in individuals without a history of coronary artery disease (CAD). Other names for this entity include, less commonly, Syndrome Y and primary CSF. Though clinically underestimated, it is a recognized phenomenon and is documented in a subset of patients with symptoms resembling acute coronary syndrome. The most common group with CSF is that of young males with a history of smoking. CSF is clinically relevant, with patients presenting with chest pain, arrhythmias, and even sudden death. CSF therefore markedly impairs patients’ quality of life. Particular treatments for CSF are not specified, though oral calcium channel blockers (CCBs) have been used successfully. Much research has been done regarding CSF, and it is thought to reflect endothelial dysfunction. Factors such as inflammation, atherosclerosis, hematological disorders, and structural components may also contribute to CSF’s pathology, though CSF’s exact cause is still not fully understood by clinicians. CSF is also distinct from conditions with similar presentations, such as post-procedural slow flow and vasospasm. Since the importance of CSF is being understood to be clinically relevant by clinicians, research is recommended to better further elucidate the pathology of this phenomenon.

## Introduction and background

Coronary slow flow (CSF) initially entered the medical literature in 1972 in a study produced by Tambe et al. [1-4]. CSF was originally visually assessed in angiographies [2]. CSF is currently characterized as a primary microvascular disease that is indicated by heightened resistance to coronary flow and slowed filling of the coronaries without associated “obstructive artery disease” [1]. Patients are diagnosed with CSF if coronary artery stenosis is below 40% and if “slow coronary flow” is assessed angiographically [3]. CSF is now defined by utilizing the “corrected thrombolysis in myocardial infarction frame” (cTFC) of greater than 27 units [3]. Thrombolysis in myocardial infarction (TIMI) scores represent the rate and diffusion of contrast through the microvasculature and are an objective method to assess slow flow as compared to visual examination [2]. Therefore, coronary angiography is the singular method used when clinicians diagnose this phenomenon [2]. Transthoracic Doppler echocardiography (TTDE) may represent a less invasive alternative and allow for follow-up over the course of the patient’s life, though further research must be done to establish TTDE as a recommended tool for CSF diagnosis [2]. 

CSF is distinct from Syndrome X, a syndrome marked by angina-like symptoms experienced by patients who have “angiographically normal coronary arteries” and is more common in postmenopausal female patients [1,5]. CSF is more prevalent in the patient population of young males with a history of smoking and has been called Syndrome Y due to the hypothesized significance of Neuropeptide Y in CSF pathogenesis [1]. It is important to note that CSF is a different clinical phenomenon from the case of slowed contrast progression during reperfusion therapy or in the setting of other entities, such as ectasia or vasospasms [1]. 

CSF is clinically very relevant, since it is associated in more than 80% of diagnosed patients with relapses of angina in frequency when patients are at rest or mixed angina due to ischemia [1,2]. About 20% of CSF patients require treatment in the coronary care unit, indicating the potential severity of the condition [2]. About 2.5% of CSF patients experience other clinical symptoms including ST-segment elevated myocardial infarction (STEMI), arrhythmias, notably ventricular, and cardiac arrest [1,3]. These symptoms may be because of enlarged QTc dispersion, which Atak et al. discovered to be heightened in CSF patients when compared to a control group of individuals with normal coronary arteries because of a decreased “minimum QTc interval” [1,6]. Studies have found that the left anterior descending (LAD) artery is most often impaired, and CSF presentations may include left ventricular dysfunction [1]. Therefore, as Aparicio et al. mentioned that CSF patients whose LAD is impaired should be even more closely monitored in follow-up [1]. These symptoms show that all CSF patients require regular and close monitoring [3]. These studies show that CSF must be treated to improve angina symptoms and prevent or reduce ventricular dysfunction [1]. Coronary slow flow phenomenon (CSFP) is generally found in young male patients with a history of smoking and metabolic syndrome, which is one of the reasons that endothelial dysfunction is a common theory for the pathogenesis underlying CSF [1]. 

CSF’s underlying etiology is not clearly understood, and researchers hypothesize that endothelial dysfunction, atherosclerotic development, and inflammatory factors, among others, may contribute to the development of CSF [3]. Since CSF’s etiology has still not been fully understood, a standard treatment guideline for CSF has not been formed [3]. Clinicians find that medications that address inflammation and endothelial dysfunction are effective, such as calcium channel blockers (CCBs), statins, and nebivolol [3]. Further research, especially in the form of robust, randomized control trials, must be done to understand CSF’s etiology and develop a standard for treatment [3].

## Review

Methods

In this narrative literature review, we studied the background research on CSF’s pathogenesis, clinical relevance and prognosis, and current recommended treatments. We searched the databases on Google Scholar and PubMed to find information on this topic using the keywords of “coronary slow flow,” “endothelial dysfunction,” “prognosis,” and “treatment" in articles up to July 2025. We chose journal articles for source material, ensuring that these were from peer-reviewed resources. We excluded any works that were not published in the English language. We read the titles and abstracts of articles to ensure that the text was relevant. After applying these criteria, we chose a total of 34 journal articles for this literature review. We then organized the discussion by themes, such as pathogenesis, treatment, and prognosis, and future research for CSF. 

Pathogenesis

Much research around CSF suggests that endothelial dysfunction is the main etiology underlying CSF. Beltrame et al. found in a study that CSF patients were more likely to present with an elevated “resting coronary vasomotor tone” [1,7]. The maintenance of coronary tone depends on the interaction between vasoconstrictors, including endothelin, and vasodilators, including nitric oxide [1]. Specifically, endothelin-1 as well as the factor called neuropeptide Y may be involved in CSF’s vascular narrowing [1]. Studies have discovered that those with CSF have heightened concentrations of endothelin-1 [3]. Other studies have also found a lowered presence of nitric oxide, such as Camsarl et al. [1,8]. In a study by Sezgin et al., they found regarding CSF patients that “endothelium dependent flow mediated dilatation” was negatively affected, possibly related to the increased homocysteine concentrations found [1,9]. Patients with CSF were also discovered to have increased concentrations of dimethylarginine, a “nitric oxide synthase inhibitor” [2]. Previous studies have also found that CSF patients have lowered levels of adiponectin and paraoxonase, both factors related to endothelial variations [2]. Therefore, researchers hypothesize that the disequilibrium of vasoconstricting and vasodilating agents produced by vascular endothelial cells contributes to the pathogenesis of CSFP [3]. 

Some studies consider CSF to be an early form of atherosclerotic development [1]. Pekdemir et al. found that atherosclerotic development was related to the heightened microvascular resistance, leading to lessened coronary “fractional flow reserve” [1,10]. Regarding CSF patients, researchers have found increased calcification of the arterial wall as well as thickening of the intima [3]. The factor named “soluble lectin-like oxidized low-density lipoprotein receptor-1 (sLOX-1)” is involved during atherosclerotic progression, as well as heightened in those affected by CSF [3]. Regarding those with CSF, Wang et al. and Yilmaz et al. found that population is likely to have metabolic syndrome, which may be related to the endothelial irregularities seen in CSF, with Wang et al. finding an increased amount of “total cholesterol, low density lipoprotein, and fasting blood glucose,” as well as an increased BMI in those with CSF [1,2,11]. Hawkins et al. found that being male and obese increased the risk of CSF, and Sanati et al. and Rouzbahani et al. discovered that hypertension was a risk factor for CSF [1,12-14]. Similar to Rouzbahani et al., Mukhopadhyay et al. assessed 80 patients in India and found BMI to be a risk factor for this clinical entity [1,14,15]. In the study by Hawkins et al., CSF patients were documented to have decreased HDL measurements, providing more support for the proposed link between CSF and atherosclerosis [1,12]. These studies add support to the hypothesis that subclinical atherosclerosis may be part of CSF’s etiology [3]. 

Further studies have investigated the involvement of small vessel disease in CSF [1,2]. Mangieri et al. studied endomyocardial biopsies and found coronary “thickening, mitochondrial abnormalities, and reduction in glycogen content”; these irregular findings indicate that small vessel abnormalities may play a role in elevated resistance to coronary blood flow [1,16]. Regarding coronary artery changes in CSF patients, studies found fibromuscular dysplasia, increased intimal growth, and degeneration [2]. Interestingly, the Beltrame et al. study found that CSFs consistently heightened tone at rest was possibly associated with the coronary sinus having a decreased oxygen concentration [1,17]. These results suggest that CSF development in the microvasculature may arise from intertwined structural as well as physiological factors [2]. 

Other more recent research has come about that questions the role of endothelial dysfunction regarding CSF’s development [1]. Kopetz et al. contrasted a group diagnosed with CSF with a normal group, and it was the first work to discover no evidence of endothelial dysfunction in CSF patients [1,18]. This study also discovered that nitric oxide concentrations were not significant in CSF development [1]. This indicates that additional agents such as endothelin-1 and factors of platelet activation warrant further study to clarify their roles in CSF’s pathophysiology [1]. Li et al. found the “adenosine phosphate-induced platelet aggregation rate” to be a CSF risk factor [1,19]. Research indicates that CSF patients had greater “red blood cell distribution width” [3]. Furthermore, CSF patients are shown to have increased “hematocrit, platelet count, and mean platelet volume” compared to controls [3]. Likewise, some researchers suggest about CSF that the inflammatory process may be linked to platelet function and hematological variations, such as Li et al.’s finding that regarding CSF, individuals had heightened C-reactive protein (CRP), interleukin 6, and uric acid [1,3,20]. Turhan et al. discovered that CSF patients had increased adhesion agents, including “vascular cell adhesion molecule-1 (VCAM-1), intercellular adhesion molecule-1 (ICAM-1), and E-selectin” [1,3,21]. CRP has been associated with heightened flow resistance and may be implicated in remodeling of the vessel walls [4]. A study by Kayapinar et al. discovered that CSF is linked to inflammatory agents such as fibrinogen and CRP [3,22]. Oylumlu et al. found the “platelet to lymphocyte ratio” to be possibly contributing to CSF [3,23]. A study by Ding et al. found that higher cytokine “plasma lipoprotein-associated phospholipase A2” concentrations were linked with increased coronary TIMI frames or slower coronary flow [3,24]. These studies’ results continue to highlight the part that platelet abnormalities and the inflammatory process may play in CSF progression [3]. 

Some studies have theorized that anatomical features may be involved in CSF’s etiology, such as Wang et al.’s finding that CSF patients’ coronaries were more highly branched and tortuous [1,2,25]. In CT angiographic studies, coronaries branching off the aorta were more acutely angled in CSF patients [1]. Researchers have proposed various theories about CSF’s etiology, ranging from endothelial dysfunction, atherosclerotic progression, platelet activation and related factors, small vessel disease, the inflammatory process, and anatomical features. Further research is needed to delve into this clinical entity to better learn about CSF and recommend standardized treatment. 

Treatment

There is no one established guideline for treatment in CSF patients [1]. Current research points towards the microvasculature as being significant in the pathogenesis of CSF, among other factors, specifically the inflammatory process and metabolic syndrome [1]. Considering the effectiveness of CCBs for vasospastic angina, which has a similar etiology regarding the microvasculature, oral CCBs have been assessed for benefits regarding CSF patients [1]. 

CCBs have been demonstrated to be effective at alleviating CSF symptoms acutely as well as in long-term patient management. Li et al.’s study with 42 patients found that diltiazem in oral form was an effective treatment for CSF patients, showing a beneficial impact on symptoms and increased blood flow in the coronaries [1,20]. For CSF, Alvarez et al. studied in 15 patients the application of another oral CCB, nifedipine, for CSF in patients who had already been successfully treated using intracoronary CCBs [1,26]. Continued treatment that extended over 13 months revealed a significant benefit in patients’ angina symptoms [1]. Mehta et al. found comparable results with administration of intracoronary nicardipine in CSF patients [1,27]. The CCB mibefradil has been documented to improve CSF symptoms in clinical use [1].

Nitrates have also been demonstrated to be beneficial at managing and alleviating CSF symptoms. Mosseri et al. considered a small sample of 6 individuals with CSF with different clinical features [1,28]. However, Mosseri et al. discovered that sublingual nitrates were effective for every patient in this study [1,28]. As per Ozdogru et al.’s study, diltiazem was more effective than intracoronary nitroglycerine [1,29]. Sani et al. and Sadamatsu et al. found comparable outcomes regarding decreases in angina symptoms with CSF patients with administration of nicorandil as compared to nitroglycerin [1,30,31]. 

Li et al., among others, have theorized regarding statin benefits for CSF patients due to its impact on dyslipidemia and endothelial factors [1,32]. In CSF patients, statins have been linked with an increase in coronary perfusion as shown by Cakmak et al., who found in a study with 97 patients that 40 mg of the statin simvastatin may be effective for reducing CSF symptoms [1,33]. The beta blocker nebivolol is reported as beneficial for CSF patients in Albayrak et al.’s study with 42 patients, which discovered that nebivolol limited angina symptoms and that it could be useful in improving the QTc dispersion found in CSF patients [1,34]. This study also demonstrated that administration of nebivolol improved the brachial artery’s “flow-mediated dilatation” [1]. 

Treatments such as dipyridamole, verapamil, adenosine, or nitroprusside have a history of being administered successfully acutely in CSF treatment [1]. A definitive treatment guideline for CSF patients can be proposed once further research is done, especially through broadly scaled clinical trials [1]. The current body of research indicates that CCBs are a very effective medication for CSF patients, but more research is necessary to thoroughly understand the etiology of CSF and outline the best treatment with strong clinical evidence [1]. 

Systemic implications

There is more to be discovered about this entity’s prognosis [1]. As a clinically distinct entity from other disorders, CSF is currently the topic of much research [1,3]. Originally thought to be a benign finding, patients with CSF were believed to have normal coronaries without cause for further clinical concern [1]. Research, including controlled trials, finds, however, that patients with CSF have a higher risk for often life-threatening ST changes as well as ventricular arrhythmias [1,3,6]. It is also not clearly understood if CSF is due to localized factors or emerges from a systemic phenomenon [1,2]. Evidence from studies, such as that of Wang et al., indicates that CSF may be a systemic condition influencing myocardial as well as peripheral vessels since changes in the brachial artery’s dilation were found [1,2]. Other researchers have found CSF patients to have an enlarged carotid intima-media thickness (IMT), implying a similar increase in the carotid vessels, lending weight to the theory that CSF pathogenesis includes the greater systemic microvasculature [1]. Furthermore, researchers discovered that CSF patients presented with a decreased cerebral flow rate compared to those without CSF [2]. Studies have found that anxious behavior and mood disturbances are linked to CSF, which may align with the theory of CSF being part of a system-wide process [1].

## Conclusions

CSF is a recognized clinical condition currently defined by specific diagnostic parameters. Identified in a small subset of angiographies, CSF holds clinical importance because it is linked to arrhythmias, angina, and even cardiac arrest. It is commonly identified among individuals diagnosed with acute coronary syndrome (ACS). Despite being clinically overlooked, the phenomenon of CSF necessitates additional research to better elucidate its pathogenesis. Current research has put forth a range of hypotheses that endothelial abnormalities, atherosclerotic development, the inflammatory response, and structural abnormalities underlie CSF’s pathogenesis. Emerging research may suggest that CSF is a phenomenon in the setting of a larger systemic disease process. Though no formal treatment regimen is outlined for CSF, CCBs have consistently been researched in many studies with documented acute and chronic symptom relief. Statins, in addition to the beta blocker nebivolol, have demonstrated potential in treating CSF, though further clinical trials will be necessary. Advancing research through further clinical trials is essential to better elucidate the etiology of CSF, develop treatment standards, and alleviate CSF’s damage to patients’ quality of life.
